# Cerebro-Spinal Meningitis

**Published:** 1872-03

**Authors:** C. F. Rodenstein

**Affiliations:** Westchester


### Miscellaneous.

### Cerebro-Spinal Meningitis.

SIR :—The rumor that epidemic cerebro-spinal meningitis has appeared in some portions of this State, and the announcement that some cases have been reported to the Board of Health, induces me to write a few lines, with the hope of calling the attention of medical practitioners, who may have the opportunity of seeing this disease, to some details of treatment, and especially to the use of the ordeal bean of calabar, which I believe has been of service to me in the management of a recent limited epidemic.

Since the beginning of November last I have treated forty cases of cerebro-spinal meningitis. Twenty-nine were well-marked typical cases. The ages of my patients ranged from 3 to 16 years. Four died, and one is not yet out of danger. As this epidemic occurred under peculiar circumstances, and presented many features which may be of interest to the profession, I propose to publish soon a full report.

The therapeutics of this disease are notoriously unsatisfactory. The mortality of reported epidemics ranges from 30 to 75 per cent. The death-rate of my cases is only 10 per cent. But their number is too small to allow any legitimate conclusion to be drawn from such limited statistics as to the value of any particular treatment. I anticipate my report, therefore by this preliminary statement, that others may not miss an opportunity of testing the value of the remedies I have employed.

Waiving for the present all discussion concerning nosology, etiology, pathology, and morbid anatomy of cerebro-spinal neningitis, I may be permitted to state that the most salient and central feature of this disease seemed to me to be an enormously increased reflex irritability of the nervous system, with an attendant paralysis of some branches of the sympatheticus. In studying my first case it occurred to me that calabar bean, which ophthalmologists use in eye-diseases dependent upon an irregular or interrupted innervation of the dioptric apparatus, and which lately has acquired considerable reputation in the treatment of various forms of tetanus, might be of use in this disease, the symptomatology of which is so largely made up by paretic and tetanoid phenomena.

At first I used the tincture of calabar bean, but afterwards I succeeded in procuring the extract. The extract was manufactured by Hazard & Caswell, 2½ drachms of which are equal to 2 ounces of the bean. I had one drachm rubbed up in glycerine and gave 2–5 drops at a dose.

I administered this drug in every case, as soon as tetanic contractions or apparent paralysis of the sphincter iridis appeared; except in two fatal cases, one of which terminated very rapidly, the other occurred before I had obtained the extract and after the tincture was exhausted.

In the great majority of cases the drug seemed to exert a favorable influence upon the symptoms which it was intended to control. In a few of the milder cases, where there was rather a contracted pupil, I administered atropia in pretty full doses, without, however, influencing the general course of the disease.

In the onset of the attack the symptoms are generally so violent, cephalalgia and rachialgia so severe and distressing, the skin and mucous membranes so congested, that depletion seems the one thing indicated, and yet I have resisted every temptation to abstract blood. I have neither bled, nor leeched, nor cupped, nor blistered. Ice in bladders to head and neck mitigated the pain and proved grateful to the patient.

Cathartics I used very moderately, not intending to produce any revulsive effect upon the bowels.

I had no reason to be dissatisfied, with this treatment, when afterward, in some of the protracted cases, a prostration set in as extreme as the precedent violence, and a degree of emaciation followed which rivalled the marasmus of the everlasting gastro-intestinal catarrh of starveling foundlings.

In the first days, or whenever, in the course of the disease, there was great restlessness, or violent jactation, I administered moderate doses of chloral hydrate, 10–15 grains, repeated as required. Morphine I gave only in one case where ice seemed unpleasant and headache was worse.

Although there is a certain rhythm and periodicity in the progress of this disease, I have seen no reason for giving quinine until after convalescence was established, and then, in combination with iron, it doubtless proves beneficial in re-establishing the vigor of the system.

In most of the cases periods of coma follow or alternate with spasms and jactations. In such cases I have found the iodide of potassium of service. But this drug must be administered with a bold and liberal hand. I have given from 2 scruples to a drachm in 24 hours; generally alternating it with the extract of calabar bean, so that my patients received every two hours either a dose of calabar (2–5 drops) or a dose of pot. iod. gr. 6–10.

Cerebro-spinal meningitis is not a self-limited disease; it is protean in its manifestations; probably more frightful in its phenomena than severe in its anatomical lesions,—at least in a majority of cases,—and offers, therefore, a hopeful field to the therapeutists, although few victories have as yet been won. To induce physicians to make new trials I have ventured to state briefly the method of treatment I have pursued; so far as its results go, I believe they are more favorable than any that have been obtained.

In general hygiene little could be done in the persent state of our knowledge as the cause of the disease. I have used carbolic acid as a disinfectant, and internally to some extent.

In concluding these lines I wish to express my gratitude to Professor Jacobi, who had the extreme kindness to visit my patients with me, and to whose profound knowledge of the diseases of childhood and skillful application of modern therapeutics I am indebted for much valuable advice. Very truly yours,

C. F. RODENSTEIN, M. D.

Westchester, March, 1872. —*The Medical Record.*

